# Microneedle Delivery of an Adjuvanted Microparticulate Vaccine Induces High Antibody Levels in Mice Vaccinated against Coronavirus

**DOI:** 10.3390/vaccines10091491

**Published:** 2022-09-07

**Authors:** Sharon Vijayanand, Smital Patil, Devyani Joshi, Ipshita Menon, Keegan Braz Gomes, Akanksha Kale, Priyal Bagwe, Shadi Yacoub, Mohammad N. Uddin, Martin J. D’Souza

**Affiliations:** Center for Drug Delivery and Research, Vaccine Nanotechnology Laboratory, College of Pharmacy, Mercer University, Atlanta, GA 30341, USA

**Keywords:** microneedles, microparticles, immunogenicity, cytotoxicity, antigen presentation, antibody response

## Abstract

This ‘proof-of-concept’ study aimed to test the microparticulate vaccine delivery system and a transdermal vaccine administration strategy using dissolving microneedles (MN). For this purpose, we formulated poly(lactic-co-glycolic) acid (PLGA) microparticles (MP) encapsulating the inactivated canine coronavirus (iCCoV), as a model antigen, along with adjuvant MP encapsulating Alhydrogel^®^ and AddaVax. We characterized the vaccine MP for size, surface charge, morphology, and encapsulation efficiency. Further, we evaluated the in vitro immunogenicity, cytotoxicity, and antigen-presentation of vaccine/adjuvant MP in murine dendritic cells (DCs). Additionally, we tested the in vivo immunogenicity of the MP vaccine in mice through MN administration. We evaluated the serum IgG, IgA, IgG1, and IgG2a responses using an enzyme-linked immunosorbent assay. The results indicate that the particulate form of the vaccine is more immunogenic than the antigen suspension in vitro. We found the vaccine/adjuvant MP to be non-cytotoxic to DCs. The expression of antigen-presenting molecules, MHC I/II, and their costimulatory molecules, CD80/40, increased with the addition of the adjuvants. Moreover, the results suggest that the MP vaccine is cross presented by the DCs. In vivo, the adjuvanted MP vaccine induced increased antibody levels in mice following vaccination and will further be assessed for its cell-mediated responses.

## 1. Introduction

Coronaviruses (CoVs) cause respiratory, systemic, or enteric diseases in various mammalian hosts resulting in mild to fatal clinical manifestations [1,2,3]. CoVs are RNA viruses named as such due to the spike-like projections on the outer membrane that are vital for the virus’s attachment and entry into the host tissue [1]. Amongst the CoVs that infect animals, the canine coronaviruses (CCoVs) have been given much importance as they widely affect dogs causing death in severe cases [2]. CCoVs exhibit 44.5% similarity to the severe acute respiratory syndrome virus (SARS-CoV) and severe acute respiratory syndrome virus-2 (SARS-CoV-2) at the nucleotide level [4]. Moreover, the pathogenesis and the early host immune responses of the canine respiratory coronavirus (CRCoV) are similar to infection caused by SARS-CoV-2 and thus have been proposed as a naturally occurring animal model of SARS-CoV-2 infection in humans [1,5]. Recent research also suggests that pets may act as intermediate hosts in the transmission of pathogenic CoVs [6]. Repeated exposure to CCoVs and other human coronaviruses (HCoVs) may produce cross-protection against the SARS-CoV-2 [6,7]. All CoVs, notably the SARS-CoV-2, which caused the recent pandemic, originated from animals, highlighting the zoonotic potential of CoVs and their ability to cause infection in humans [1,4]. Moreover, due to the structural resemblance to other HCoVs, the chemically inactivated CCoV (iCCoV) was used in this study as a model to test the microparticulate delivery of the vaccine antigen [4,6].

Microparticulate formulations for vaccines have been researched extensively and result in the increased immunogenicity of the antigen [8,9,10,11]. The increased immunogenicity may be attributed to the ease of recognition of the particulate form of the vaccine due to the optimum size and shape of the MP, resulting in increased cellular uptake by the antigen-presenting cells (APCs) such as dendritic cells (DCs) and macrophages [12,13,14]. The MP in the 100–3000 nm size range is better engulfed and presented to the T cells by the circulating APCs [12,13]. Soluble antigens are less-immunogenic due to their small size and are not easily recognized as foreign by the host immune cells [15]. However, by encapsulating such antigens in a biodegradable polymer matrix, the immunogenicity of the antigen can be significantly increased [16]. In this proof-of-concept study, Poly (lactic-co-glycolic acid) (PLGA) was used to encapsulate the iCCoV antigen into polymeric MP via a double emulsion probe homogenization method to formulate MP of size less than 3000 nm.

To further increase the antigen’s immunogenicity and potentiate the vaccine response, adjuvants are typically used in combination with the vaccine. Alhydrogel^®^ has been well-documented to recruit APCs, thereby improving the antigen uptake and presentation to T cells by the APCs [17,18,19,20,21]. Primarily, Alhydrogel^®^ works by forming a depot at the administration site, which helps maintain the physical and chemical properties of the vaccine antigen, also known as the repository effect [22]. Previously, it has been demonstrated that aluminum-based adjuvants induce a Th2 type of response and production of cytokines; however, they fail to stimulate Th1 type responses such as IFNγ production and IgG2a secretion [23]. The restricted range of immune responses induced by this adjuvant poses a significant problem during vaccine development [23]. AddaVax™, a squalene-based oil-in-water (*w*/*o*) nano-emulsion, is known to promote Th1 and Th2 type responses [24,25,26]. AddaVax™, similar to MF59, induces proinflammatory cytokines and chemokines, associated with improved recruitment, activation, and maturation of APCs at the injection site [24]. Alhydrogel^®^ and AddaVax™ are both used as adjuvants in licensed vaccines as they have shown to improve the immunogenicity of the vaccine formulation [27].

To minimize pain during immunization and to improve the vaccination rate, Microneedles (MN) for vaccination are highly desirable. The skin’s immune-rich epidermal and dermal layer is an attractive site for vaccine administration [28,29]. It consists of Langerhans cells and circulating dendritic cells capable of binding to and eliminating the invading pathogen [28,29,30,31,32]. These immune cells digest the vaccine antigen and present it on their surface via the major histocompatibility complex (MHC) class I and class II molecules, which are then recognized by T-cells [33,34]. Immunization via the skin using microneedles (MN) can be an effective vaccine delivery strategy for infectious diseases such as COVID-19 and Influenza that require frequent immunizations due to the emergence of mutant strains. Moreover, MN can be self-administered, reducing the need for trained pharmacists, nurses, and physicians for vaccine administration and may also increase the vaccination rate [35].

This proof-of-concept study demonstrates the use of polymeric microparticles (MP) as an effective antigen carrier system and dissolving microneedles for intradermal vaccine administration. The concept of MP vaccine administered using dissolving MNs has not been explored previously. In this study, we formulated PLGA MP encapsulating the iCCoV as a model antigen which was then characterized and evaluated for its in vitro immunogenicity and cytotoxicity. The adjuvant MP, Alhydrogel^®^ and AddaVax™, were screened in vitro individually and in combination for their ability to improve the immunogenicity of the antigen. We also assessed the cytotoxicity of the adjuvants in vitro. We quantified the levels of MHC I and MHC II and their corresponding co-stimulatory molecule expressions on the surface of DCs following stimulation by the MP. Finally, we evaluated the in vivo immunogenicity of the iCCoV MP with or without adjuvants by measuring the antibody response after MN administration.

## 2. Materials and Methods

### 2.1. Materials

PLGA (75:25) was purchased from Evonik Industries (Essen, Germany). Pierce Micro BCA™ (bicinchoninic acid) Assay Kit was purchased from Thermo Fisher Scientific (Waltham, MA, USA). Sodium hyaluronate (HA) (100 kDa) was purchased from Lifecore Biomedical (Chaska, MN, USA). Trehalose dihydrate, polyvinyl alcohol (PVA) (Avg Mol Wt. 30,000–70,000), and dichloromethane (DCM) were purchased from Sigma-Aldrich (St. Louis, MO, USA). The 8 × 8 array silicone microneedle templates were purchased from Micropoint Technologies (Singapore). Murine dendritic cells (DCs), DC2.4 were received as a kind gift from Dr. Kenneth L. Rock (Dana-Farber Cancer Institute, Inc., Boston, MA, USA). The iCCoV antigen was obtained from BEI Resources (NIAID, NIH: Canine Coronavirus, UCD1, Chemically Inactivated, NR-869). Fluorescein isothiocyanate (FITC) and Allophycocyanin labeled anti-mouse MHC I, MHC II, CD40, and CD80 antibodies for flow cytometric analysis were purchased from eBioscience laboratories (San Diego, CA, USA). Dulbecco’s Modified Eagle’s Medium (DMEM), fetal bovine serum (FBS), penicillin/streptomycin, and non-essential amino acids were obtained from American Type Culture Collection (ATCC) (Manassas, VA, USA). Alhydrogel^®^ (alum) and AddaVax™ were purchased from InvivoGen (San Diego, CA, USA). Six–8-week-old Swiss Webster mice were purchased from Charles River Laboratories (Wilmington, MA, USA). HRP-tagged secondary goat anti-mouse antibodies IgG, IgG1, IgG2a, and IgA were procured from Invitrogen (Rockford, IL, USA).

### 2.2. Methods

#### 2.2.1. Formulation of Vaccine Microparticles (MP)

The iCCoV vaccine MP and adjuvants MP (Alhydrogel^®^ MP and AddaVax™ MP) were formulated using a double emulsion (W_1_/O_1_/W_2_) with solvent evaporation method described previously [35]. Briefly, the vaccine antigen (1% loading) in an aqueous phosphate buffer (pH 7.4) (W_1_) was added to a 2% *w*/*v* solution of PLGA in DCM (O_1_), and probe homogenized using a 30 s on/30 sec off cycle at 17,000 RPM for 2 min to form the primary emulsion (W_1_/O_1_). The primary emulsion was then added to a 0.1% *w*/*v* solution of PVA in deionized water (W_2_) and probe homogenized at 17,000 RPM for 2 min to form the double emulsion (W_1_/O_1_/W_2_). The final emulsion was stirred for 5 h at 500 RPM to remove the residual DCM. The MP were washed in deionized water at 17,000 RPM for 10 min to remove the excess PVA and concentrate the MP into a pellet. The pellet was resuspended in 1 mL using 2% *w*/*v* trehalose solution as a cryoprotectant. The formulations were then transferred to pre-weighed glass vials and lyophilized.

#### 2.2.2. Percentage Recovery Yield and Characterization of MP

The percent recovery yield of the lyophilized product was calculated using the following formula [11,36].

$$Percent\;Recovery\;yield=\frac{Weight\;of\;lyophilized\;MP\times 100}{Weight\;of\;all\;ingredients\;in\;the\;formulation}$$

Qualitative analysis was performed by observing the vaccine MP under the scanning electron microscope for characteristic features such as size and shape. Quantitatively, the MP were characterized for their size, charge and poly-dispersity index (PDI). In brief, 1 mg of the particles were suspended in 1 mL of deionized water. Next, 50 μL of the stock was diluted in 1 mL of deionized water and transferred to a cuvette. The size of the MP, surface charge, and PDI were measured using a Zetasizer Nano ZS (Malvern Pananalytical, Westborough, MA, USA).

#### 2.2.3. Percentage Encapsulation Efficiency (% EE)

The encapsulation efficiency (EE) of the iCCoV antigen in the MP was assessed using a micro-Bicinchoninic acid (BCA) assay as per the manufacturer’s instructions and described previously [35,37]. Briefly, 5 mg of the vaccine MP were weighed, and DCM was added to dissolve the PLGA matrix. The antigen was concentrated into a pellet by centrifugation and the supernatant containing the dissolved PLGA was discarded. The residual DCM was removed by vacuum evaporation for 30 min. The extracted antigen was resuspended in 1 mL PBS and analyzed using a BCA assay. A standard curve was plotted, and the concentration per ml (conc/mL) was determined from the standard curve. The % encapsulation efficiency (% EE) was calculated using the following formula.

$$\%\;EE=\frac{Practical\;concentration\;of\;antigen\;in\;5\;mg\;of\;MP\times 100}{Theoretical\;concentration\;of\;antigen\;in\;5\;mg\;of\;MP}$$

#### 2.2.4. In Vitro Immunogenicity Assessment of Vaccine and Adjuvant MP

When stimulated by any external pathogen, APCs such as the DCs and macrophages produce nitric oxide (NO) and its metabolites nitrite and nitrate, which play an essential role in non-specific immunity. Here, an increase in nitrite production was assessed as a marker for in vitro immunogenicity of the antigen/adjuvant in the particulate form. The nitrite produced by the DCs was quantified using Griess’ assay as described previously [37,38]. First, murine DCs were plated at 3 × 10^4^ cells/well density. The cells/well were pulsed with antigen/adjuvant MP equivalent to the antigen/adjuvant dose for each group and incubated at 37 °C for 24 h. The groups included No Treatment (-ve control), iCCoV suspension (2 μg), iCCoV MP (2 μg), Alhydrogel^®^ MP (3 μg), AddaVax™ (0.5 μg), iCCoV MP (2 μg) + Alhydrogel^®^ MP (3 μg), iCCoV MP (2 μg) + AddaVax™ (0.5 μg) MP, iCCoV MP (2 μg) + Alhydrogel^®^ MP (3 μg) + AddaVax™ (0.5 μg) MP. After the exposure period, the supernatants (50 μL/well) were transferred to a fresh 96-well plate, and 50 μL of 1% sulfanilamide in 5% phosphoric acid was added to each well and incubated for 5–10 min at room temperature protected from light. Then, 50 μL of 0.1% NED (N-1-naphthyl ethylenediamine dihydrochloride) in deionized water was added to each well and incubated for 5–10 min at room temperature protected from light. The appearance of a purple/magenta color indicates the presence of nitrite. The absorbance was then read at 540 nm using a BioTek Synergy H1 plate reader (BIO-TEK Instruments, Winooski, VT, USA). The nitrite content was quantified using a sodium nitrite standard curve.

#### 2.2.5. In Vitro Cytotoxicity Assessment of Vaccine and Adjuvant MP

The in vitro cytotoxicity of the iCCoV MP and adjuvants MP was tested in DCs using an MTT (3-(4,5-Dimethylthiazol-2-yl)-2,5-diphenyltetrazolium bromide) assay as described previously [37,38]. Briefly, DCs were plated in a 96-well plate at the density of 1 × 10^4^ cells/well [39]. Two-fold serial dilutions of the vaccine MP ranging from 31.25 ug/mL to 500 ug/mL were prepared in cDMEM (DMEM high glucose medium with 2 mM L-glut, sodium pyruvate, 10% FBS, and 1% Penicillin-Streptomycin). Next, 100 μL of each concentration was added in triplicates to the wells and incubated for 24 h at 37 °C. The supernatants were then discarded, 10 μL of the MTT reagent (5 mg/mL in PBS) was added to all the wells and the volume/well was made up to 100 μL with cDMEM. The plate was incubated at 37 °C for 4 h, protected from light. Next, 100 μL of dimethyl sulfoxide (DMSO) was added to all the wells, and the absorbance was measured at 570 nm using a plate reader.

#### 2.2.6. Determining the Expression of Antigen-Presenting Molecules and Their Co-Stimulatory Molecules

Once administered, the vaccine antigen is recognized and engulfed by the circulating APCs via phagocytosis, which is then processed and presented on the surface of these cells via the MHC Class I or MHC class II pathways [34,37,40]. The expression of the antigen-presenting molecules, MHC I/II, and their co-stimulatory molecules, CD80/40, on the surface of DCs was assessed using fluorescent markers through flow cytometry analysis described previously [36,37,38,41]. For this purpose, murine DCs were plated in a 48-well plate at 3 × 10^4^ cells/well density. The cells/well were pulsed with antigen/adjuvant MP equivalent to the antigen/adjuvant dose for each group and incubated at 37 °C for 24 h. The groups included No Treatment (-ve control), iCCoV suspension (2 μg), iCCoV MP (2 μg), and iCCoV MP (2 μg) + Alhydrogel^®^ MP (3 μg) + AddaVax™ (0.5 μg) MP. The cells were stained with FITC-labeled anti-mouse MHC I or MHC II and Allophycocyanin labeled anti-mouse CD40 or CD80 for 1 h at 4 °C and kept protected from light. The stained cells were washed three times with phosphate-buffered saline (PBS), after which the fluorescence intensity was quantified using a BD Accuri C6 Plus flow cytometer (BD Bioscience, San Jose, CA, USA).

#### 2.2.7. Preparation and Characterization of Vaccine-Loaded Quick Dissolving MNs

**Figure 1:**
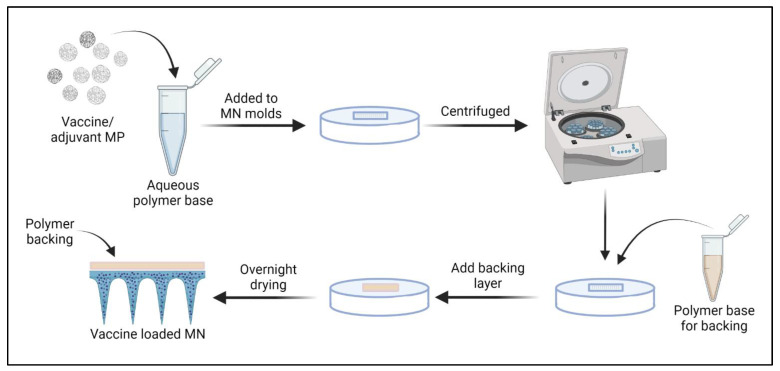
**Preparation of vaccine-loaded MN**: Vaccine/adjuvant MP were mixed with the polymer gel base, added to MN molds, and centrifuged to form the MNs. A backing layer of 10% HA was added, and the needles were allowed to dry overnight. The prepared MN were then removed and used for in vivo immunization. Image was created using Biorender.com (accessed on 3 August 2022).

The vaccine-loaded MN were prepared using a spin-casting method as described previously [35]. First, the HA-trehalose gel was prepared by combining 10% *w***/***v* HA and 5% *w*/*v* trehalose in deionized water. The vaccine MP required for each patch was calculated based on the antigen dose/mouse. The number of particles to be weighed was calculated considering the % EE. Vaccine and adjuvant MP were dispersed into the hyaluronic acid-trehalose gel. Next, 25 mg of gel was added to each pre-weighed poly dimethyl siloxane (PDMS) MN mold (8 × 8 array) and centrifuged at 4000 rpm for 15 min at 15 °C to form the needles. The MN were allowed to dry overnight in the molds, and a layer of 10% HA gel was added to the molds to serve as the backing (Figure 1). The final MN were observed under the scanning electron microscope for morphology/appearance.

#### 2.2.8. In Vivo Immunization Using MN

**Figure 2:**
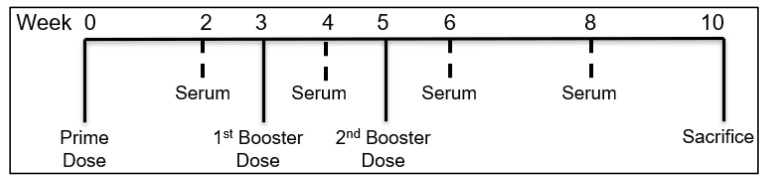
**Timeline of in vivo immunization study in mice.** The mice were vaccinated in three doses at W0, W3, and W5. The sera of the mice were collected and assessed for their antibody levels at W2, W4, W6, and W8. The mice were then sacrificed at W10.

The iCCoV MP with or without adjuvants was tested in vivo to assess its ability to increase the serum antibody levels in mice following immunization using MN. For this purpose, 6–8-week-old male Swiss Webster (CFW) mice (*n* = 4) were immunized via the skin with the vaccine-loaded MN patches. The testing was carried out as per approved Mercer University IACUC protocol. The antigen dose/mouse was 20 μg of iCCoV, the Alhydrogel^®^ dose/mouse was 30 μg, and the AddaVax™ dose/mouse was 5 μg (Table 1). The animals were divided into the following treatment groups: No Treatment group which served as a control, iCCoV suspension, iCCoV MP, and iCCoV MP + Alhydrogel^®^ MP + AddaVax™ MP group (Table 1). All treatments were given via the intradermal route using dissolving MNs. One day before each immunization, a 2 cm × 2 cm patch of the hair was removed using a depilatory cream from the back of anesthetized mice (inhalational Isoflurane) to allow for microneedle application. The animals were immunized with one prime dose at week 0 and two booster doses at weeks 3 and 5. The mice sera were collected bi-weekly and tested for the antibody levels. The animals were sacrificed at week 10 (Figure 2).

#### 2.2.9. Assessment of Serum Antibody Levels following MN Vaccination

Serum IgG, IgG1, IgG2a, and IgA levels were assessed using an enzyme-linked immunosorbent assay (ELISA). Briefly, high-binding 96-well plates (MICROLON^®^, High binding, 96 well plate, Greiner bio one) were coated with 50 μL/well of the iCCoV antigen (0.2 μg/well) in carbonate buffer (pH = 9.6) and incubated overnight at 4 °C. The plates were then washed three times with 200 μL of 0.01% Tween 20 in phosphate-buffered saline (T-PBS) solution and blocked with 3% Bovine Serum Albumin (BSA) T-PBS (50 μL/well) for 3 h at 37 °C. The plates were washed, and 50 μL of the diluted serum samples (dilution ratio 1:50) were added to the wells and incubated overnight at 4 °C. Following incubation, the plates were rewashed, and 50 μL of the HRP-tagged secondary goat anti-mouse IgG, IgG1, IgG2a, or IgA antibodies (dilution range 1:2000 to 1:4000) were added to each well and incubated for 90 min at 37 °C. Next, the plates were washed to remove the excess unbound secondary antibodies, and 50 μL of the TMB (3,3′,5,5″ -tetramethyl benzidine) substrate reagent (BD OptEIA™, BD Biosciences, San Jose, CA, USA) was added to each well and incubated for 10 min at room temperature. 50 μL of 0.3 M H_2_SO_4_ was added to each well to stop the reaction. The absorbance of plates was read at 450 nm using a plate reader.

#### 2.2.10. Statistical Analysis

All statistical analyses were conducted using the GraphPad Prism 9.2.0 software (GraphPad Software, San Diego, CA, USA). All experiments were performed in triplicate unless stated explicitly. For normally distributed data with independent groups, One-way ANOVA was used. For dependent groups, Two-way ANOVA was used. To compare means, post hoc Tukey test (to compare between means) or post hoc Dunnett test (to compare means to control) was used. The following *p* values were used, *p* > 0.05 (ns—non-significant), *p* ≤ 0.05 (*), *p* ≤ 0.01 (**), *p* ≤ 0.001 (***), and *p* ≤ 0.0001 (****). A *p*-value of <0.05 is considered statistically significant. Data is expressed as Mean ± Standard Error Mean (SEM).

## 3. Results

### 3.1. Percentage Recovery Yield and Characterization of MP

**Figure 3:**
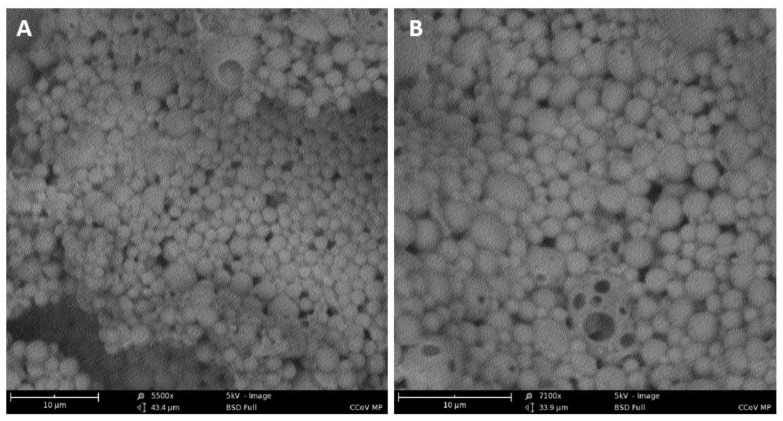
**Characterization of MP**. A. Scanning electron microscope image of MP at a magnification of 5500× (**A**) and 7100× (**B**). The MP were spherical with smooth surfaces.

The percentage yield of all the MP preparations were greater than 90% *w*/*w* (Table 2). Scanning electron microscope images reveal that the iCCoV MP are spherical with smooth surfaces (Figure 3A,B).

The average size of the iCCoV MP and adjuvants MP was 800–1600 nm ± 200–260 nm (Mean ± SD) as measured with a Malvern Zetasizer (Malvern Panalytical Ltd., Worcestershire, UK). The zeta potential (surface charge) expressed as Mean ± SD was found to be about −15.4 ± 2.31 mV for the iCCoV MP, −12.5 ± 0.252 mV for the AddaVax™ MP, and +12 ± 2.65 mV for the Alhydrogel^®^ MP. The PDI was in the range of 0.7 to 0.9 for both the iCCoV MP and adjuvant MP (Table 2).

### 3.2. Percentage Encapsulation Efficiency (% EE)

The % EE of the vaccine MP was calculated to be 91.7% using the BCA assay. The % EE represents the amount of antigen successfully encapsulated into the polymer matrix.

### 3.3. In Vitro Immunogenicity of Vaccine and Adjuvant MP

**Figure 4:**
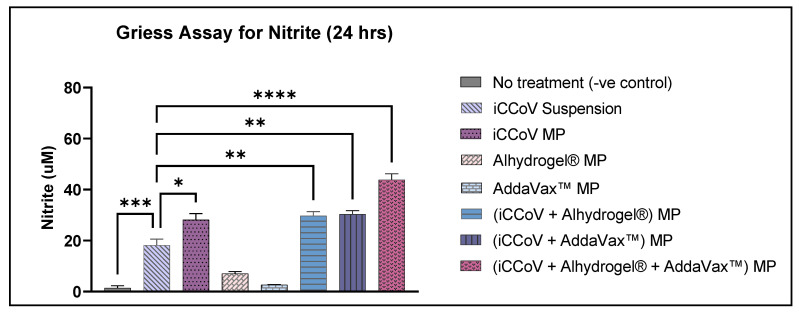
**Nitrite release from DCs following treatment with MP**. Cell density was adjusted to 3 × 10^4^ cells/well and then treated with the following groups for 24 h: No Treatment (-ve control), iCCoV suspension (2 μg), iCCoV MP (2 μg), Alhydrogel^®^ MP (3 μg), AddaVax™ (0.5 μg), iCCoV MP (2 μg) + Alhydrogel^®^ MP (3 μg), iCCoV MP (2 μg) + AddaVax™ (0.5 μg) MP, iCCoV MP (2 μg) + Alhydrogel^®^ MP (2 μg) + AddaVax™ (0.5 μg) MP. The nitrite release in the supernatant was assessed using the Griess assay. Data are expressed as mean ± SEM (*n* = 3), One-way ANOVA test, Post hoc Dunnett’s multiple comparisons test, * *p* ≤ 0.05, ** *p* ≤ 0.01, *** *p* ≤ 0.001, and **** *p* ≤ 0.0001.

The NO produced by the DCs was determined from the concentration of its oxidative product, nitrite. The cells treated with iCCoV MP, (iCCoV + Alhydrogel^®^) MP, (iCCoV + AddaVax™) MP, and (iCCoV + Alhydrogel^®^ + AddaVax™) MP released significantly higher levels of nitrite as compared to the iCCoV suspension group. The cells treated with Alhydrogel^®^ MP and AddaVax™ MP did not induce a significant nitrite release (Figure 4).

### 3.4. In Vitro Cytotoxicity of Vaccine and Adjuvant MP

**Figure 5:**
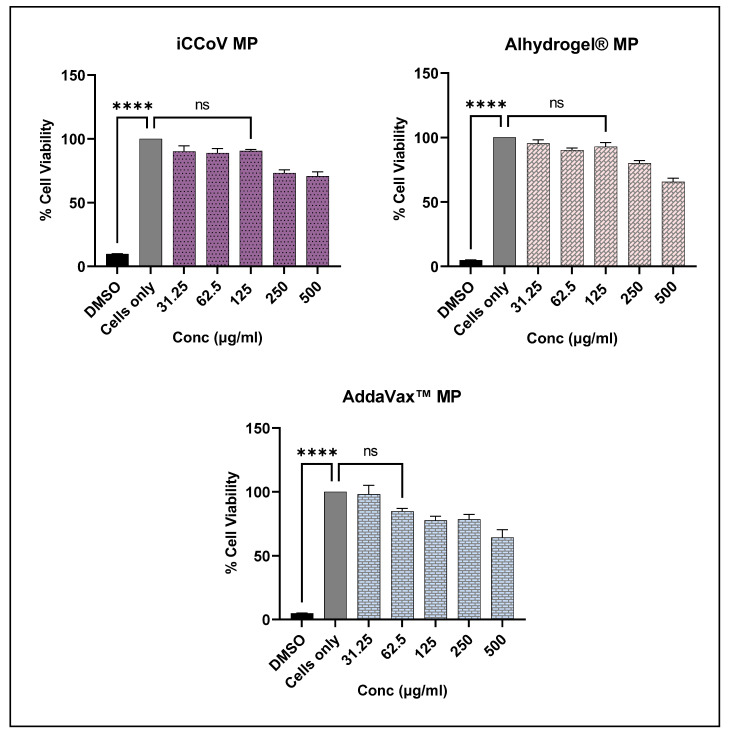
Percent Viability of DCs pulsed with varying concentrations of iCCoV MP, Alhydrogel^®^ MP, and AddaVax™ MP as measured using MTT assay. Cell density was adjusted to 1 × 10^4^ cells/well. The cells were exposed to two-fold serial dilutions of the corresponding MP groups ranging from 31.25 ug/mL to 500 ug/mL in cDMEM (100 μL/well) for 24 h. DMSO (50 μL) was used as a -ve control, and cells only were used as a +ve control. Data are expressed as mean ± SEM (*n* = 3). One-way ANOVA test, Post hoc Dunnett’s multiple comparison test, **** *p* ≤ 0.0001, ns, non-significant.

The cytotoxicity of the iCCoV MP, Alhydrogel^®^ MP, and AddaVax™ MP was tested in vitro by performing the MTT assay. The results showed that the iCCoV MP and the Alhydrogel^®^ MP were non-cytotoxic to the DCs up to 125 μg/mL. Further increase in the concentration caused a reduction in the cell viability (250 μg/mL and 500 μg/mL). Addavax™ MP was non-cytotoxic up to a concentration of 62.5 μg/mL. Higher concentrations caused a reduction in cell viability (125 μg/mL to 500 μg/mL). DMSO, used as the positive control, resulted in a significant decrease in cell viability (Figure 5).

### 3.5. Expression of Antigen-Presenting Molecules and Their Co-Stimulatory Molecules

**Figure 6:**
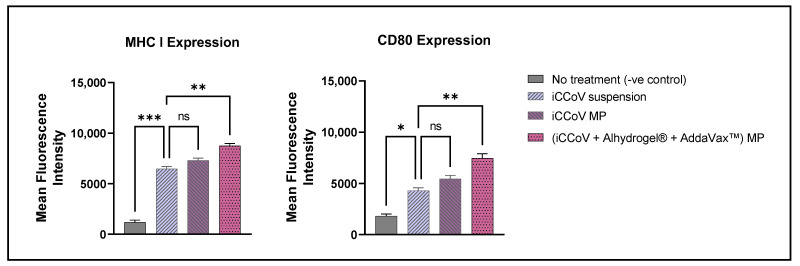
**Expression of MHC I and CD80 on the surface of murine DCs pulsed with various groups**. Cells were plated at a density of 3 × 10^4^ cells/well. The cells were exposed to the following treatment groups for 24 h: No Treatment (-ve control), iCCoV suspension (2 μg), iCCoV MP (2 μg), and iCCoV MP (2 μg) + Alhydrogel^®^ MP (3 μg) + AddaVax™ (0.5 μg) MP. Data are expressed as Mean ± SEM (*n* = 3), One-way ANOVA test, Post hoc Dunnett’s multiple comparisons test, ns, non-significant, * *p* ≤ 0.05, ** *p* ≤ 0.01, *** *p* ≤ 0.001.

The expression of the antigen-presenting molecules, MHC I and MHC II, and their co-stimulatory molecules, CD80 and CD40, respectively, on the surface of DCs, was assessed using a BD Accuri C6 Plus flow cytometer (San Jose, CA, USA). The cells treated with just the iCCoV MP expressed increased MHC I and CD80 expression; however, there was no significant difference compared to the iCCoV suspension group (Figure 6). The cells treated with the (iCCoV + Alhydrogel^®^ + AddaVax™) MP resulted in a significantly higher expression of MHC I and CD80 than the iCCoV suspension group (Figure 6).

**Figure 7:**
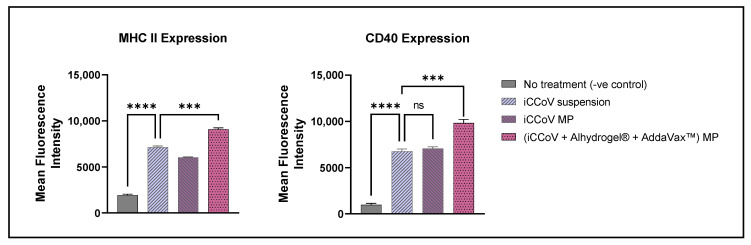
**Expression of MHC II and CD40 on the surface of murine DCs pulsed with various groups.** Cells were plated at a density of 3 × 10^4^ cells/well. The cells were exposed to the following treatment groups for 24 h: No Treatment (-ve control), iCCoV suspension (2 μg), iCCoV MP (2 μg), and iCCoV MP (2 μg) + Alhydrogel^®^ MP (3 μg) + AddaVax™ (0.5 μg) MP. Data are expressed as Mean ± SEM (*n* = 3), One-way ANOVA test, Post hoc Dunnett’s multiple comparisons test, ns, non-significant, *** *p* ≤ 0.001, and **** *p* ≤ 0.0001.

Likewise, the MHC II and CD40 expression in the cells treated with (iCCoV + Alhydrogel^®^ + AddaVax™) MP group was significantly higher than in the iCCoV suspension group (Figure 7). There was no significant difference in the MHC II and CD40 expression in the cells treated with the iCCoV MP compared to the iCCoV suspension (Figure 7). Thus, the (iCCoV + Alhydrogel^®^ + AddaVax™) MP group resulted in increased antigen presentation than the iCCoV suspension group in vitro.

### 3.6. Characterization of MN

**Figure 8:**
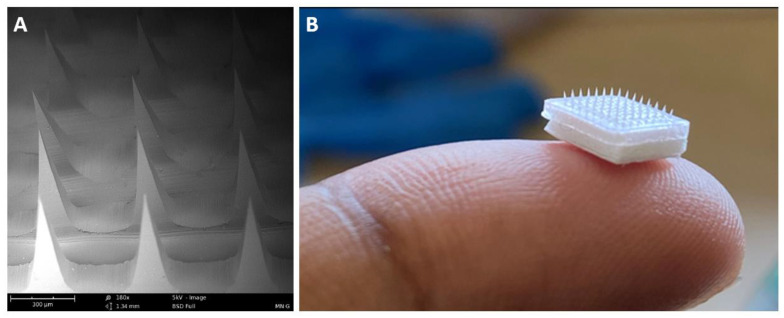
**Characterization of MN**. Scanning electron microscope and Digital images of microneedle arrays. Scanning electron microscope image of MN patch at a magnification of 180× (**A**). Digital image of MN depicting its size when placed on a fingertip (**B**).

The MNs were observed under the scanning electron microscope. The scanning electron microscope images confirmed the formation of sharp needles approximately 520 μm in length (Figure 8). A detailed optimization and characterization study of the dissolving MNs from our group was published recently [35].

### 3.7. Assessment of Serum Antibody Levels Using ELISA

**Figure 9:**
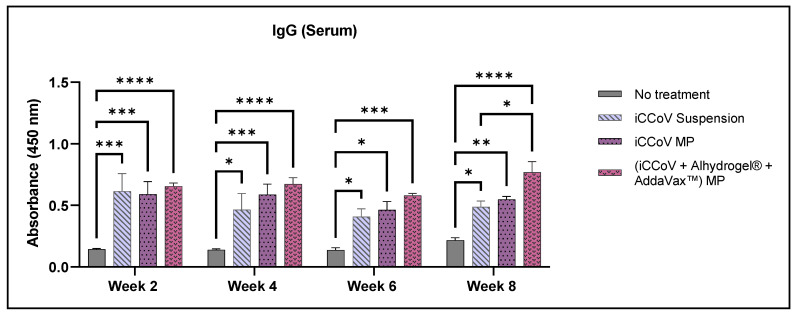
**Assessment of serum IgG antibody levels in mice.** (Absorbance at 450 nm). Responses obtained are compared to the naïve group (No treatment control) and the iCCoV suspension group. Data are expressed as mean ± SEM, *n* = 4 mice, Two-way ANOVA, Post hoc Tukey’s multiple comparisons test. ns, non-significant, * *p* ≤ 0.05, ** *p* ≤ 0.01, *** *p* ≤ 0.001, **** *p* ≤ 0.0001.

The antibody response obtained following the MN administration of the iCCoV antigen suspension, iCCoV MP, and (iCCoV + Alhydrogel^®^ + AddaVax™) MP was tested in 6–8-week-old Swiss Webster mice. IgG, IgA, IgG1, and IgG2a antibody levels in vaccinated mice sera were assessed using ELISA. The mean antibody responses obtained were compared to the No treatment group and the iCCoV suspension group. All groups elicited a significantly higher IgG response than the No treatment group during weeks 2, 4, 6, and 8. Compared to the iCCoV suspension group, the IgG responses of the (iCCoV + Alhydrogel^®^ + AddaVax™) MP group were significantly higher only during week 8 (Figure 9).

**Figure 10:**
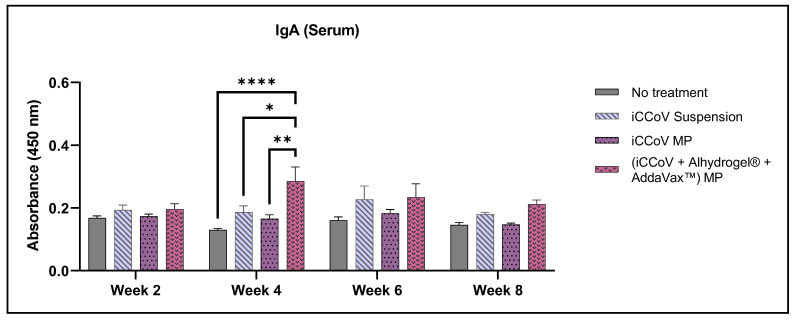
**Assessment of serum IgA antibody levels in mice**. (Absorbance at 450 nm). Responses obtained are compared to the naïve group (No treatment control) and the iCCoV suspension group. Data are expressed as mean ± SEM, *n* = 4 mice, Two-way ANOVA, Post hoc Tukey’s multiple comparisons test. * *p* ≤ 0.05, ** *p* ≤ 0.01, **** *p* ≤ 0.0001.

The IgA levels of the (iCCoV + Alhydrogel^®^ + AddaVax™) MP group peaked during week 2 and were significantly higher than the No treatment group and iCCoV suspension group (Figure 10).

**Figure 11:**
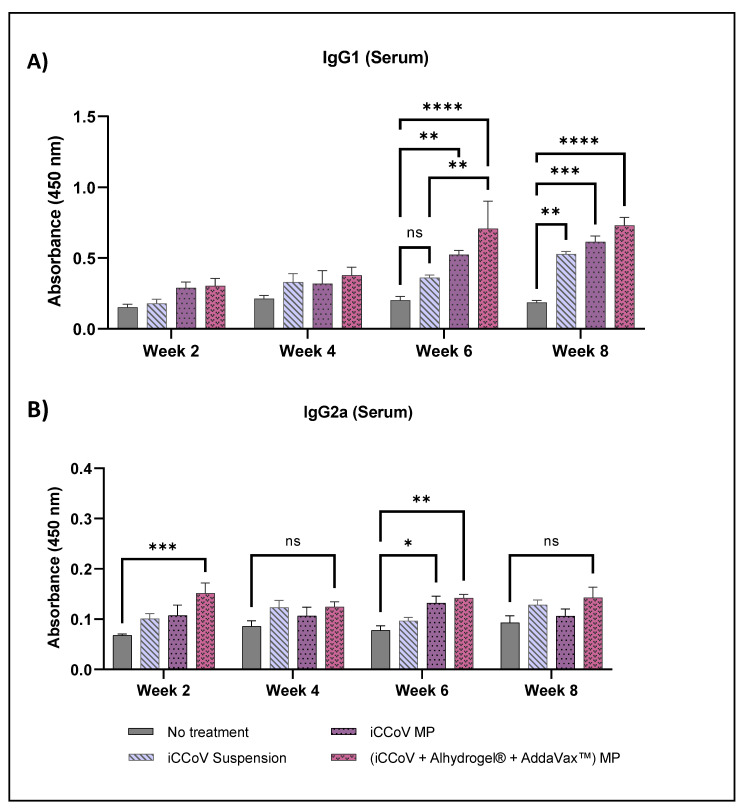
Assessment of serum IgG1 and IgG2a antibody levels in mice. Serum IgG1 Levels (**A**) and serum IgG2a levels (**B**). (Absorbance at 450 nm). Responses obtained are compared to the naïve group (No treatment control) and the iCCoV suspension group. Data are expressed as mean ± SEM, *n* = 4 mice, Two-way ANOVA, Post hoc Tukey’s multiple comparisons test. ns, non-significant, * *p* ≤ 0.05, ** *p* ≤ 0.01, *** *p* ≤ 0.001, **** *p* ≤ 0.0001.

IgG subtyping revealed that the IgG1 level of the iCCoV suspension group increased significantly only during week 8 compared to the No treatment group. However, IgG1 levels of the iCCoV MP and (iCCoV + Alhydrogel^®^ + AddaVax™) MP group were significantly higher than the No treatment groups for weeks 6 and 8 (Figure 11A). Serum IgG2a levels of the (iCCoV + Alhydrogel^®^ + AddaVax™) MP group were significantly higher during weak 2 and 6 than in the No treatment group (Figure 11B). Thus, the overall serum antibody responses were higher for the mice receiving (iCCoV + Alhydrogel^®^ + AddaVax™) MP than for the No treatment group.

## 4. Discussion

We formulated, characterized, and tested a microparticulate delivery system using iCCoV as a model antigen in this study. Further, we formulated, characterized, and tested dissolving microneedles for the MP vaccine administration and assessed the serum antibody responses induced by vaccination in mice.

Polymeric MP are a very versatile delivery system, often used to deliver therapeutic proteins and vaccine antigens [42]. The polymeric particles prevent antigen degradation by enzymes and other tissue fluids and facilitate the vaccine’s cellular uptake [37,42]. Previously, we have shown that PLGA encapsulation of various antigens using a double emulsion method results in increased cellular uptake and antigen presentation [35,37,42]. We formulated the iCCoV MP and the adjuvants, Alhydrogel^®^ MP and AddaVax™ MP, using PLGA to encapsulate the antigen/adjuvant. The formulations resulted in spherical MP with high product yield (>90% *w*/*w*) and MP size ranging from 800–1600 nm. It has been reported that MP in the size range of 1 to 3 μm are easily recognized and engulfed by circulating APCs [14,41]. The charge of the antigen/adjuvant MP varied depending on the encapsulated material. The Alhydrogel^®^ MP were positively charged, whereas the iCCoV MP and the AddaVax™ MP were negatively charged. The positive charge of the Alhydrogel^®^ MP may be attributed to the positive charge of aluminum hydroxide itself. Generally, a higher positive or negative charge is preferred for colloidal suspensions to prevent agglomeration of the particles when suspended [43]. The % EE of the iCCoV antigen in the PLGA MP was 91.7% *w*/*w*, indicating that the PLGA MP can be used as an effective antigen carrier system. 

Utilizing such a carrier system renders the antigen more immunogenic, capable of producing a heightened immune response [10]. Therefore, we tested this hypothesis by performing an in vitro Griess’s nitrite release assay using DCs. The circulating and tissue-resident immune cells, such as DCs and macrophages, release NO when they encounter an invading pathogen [44,45]. NO, and its metabolic products, nitrite, and nitrate play a significant role in generating non-specific immune responses, including the release of cytokines and recruitment of APCs [46,47]. The results indicate that the particulate form of the vaccine was observed to be more immunogenic than the iCCoV antigen suspension. The adjuvants MP were non-immunogenic or displayed negligible immunogenicity on their own but showed improved responses when combined with the iCCoV MP. Adding a single adjuvant to the iCCoV MP produced a response equivalent to the unadjuvanted iCCoV MP; however, when the two adjuvants were combined with the iCCoV MP, the nitrite release was much higher. Thus, the combination of Alhydrogel^®^ MP and AddaVax™ MP potentiated the immunogenicity of the vaccine MP. 

For a vaccine to be considered safe, it must be non-toxic to cells [42]. The cell cytotoxicity study suggested that the vaccine MP is safe at various concentrations. The percent cell viability was quantified based on the ability of metabolically active cells (live cells) to reduce MTT salts to formazan crystals [48]. For this purpose, we tested concentrations ranging from 31.25 μg/mL to 500 μg/mL. The vaccine was found safe and resulted in no significant reduction in cell viability at a concentration of up to 125 μg/mL in vitro. This finding can be further corroborated by assessing the safety in vivo, which is outside the scope of this study.

An effective vaccine should induce antigen-specific immune responses [19,32]. Such a response is generated when the antigen is engulfed and presented to the T cells by the APCs [34]. Thus, the APCs serve as a link that bridges the non-specific innate immunity and the antigen-specific adaptive immunity [49]. Therefore, antigen presentation via the MHC I and II is vital for inducing a robust immune response. Further, activation of T cells requires two signals: the binding of the MHCI/II to the TCR and the binding of the co-stimulatory molecules CD40/80 to CD28 of the T cell [50,51]. We observed that the adjuvanted vaccine MP induced a higher expression of the MHCI/II and its co-stimulatory molecules than the iCCoV suspension suggesting that APCs better present the adjuvanted microparticulate vaccine. 

Moreover, when T cells are activated, they can induce either a Th1 or Th2 type immune response depending on whether the APCs present the antigen via the MHC I or II pathway [40]. A significant consideration for viral vaccines is the activation of cellular immune responses, particularly the generation of cytotoxic T lymphocytes (CTLs) capable of recognizing and killing virus-infected cells [52]. CTLs are activated when the APCs present the antigen via MHC I, representing a Th1 type of immune response [34]. Whether a vaccine generates a Th1 or Th2 type of immune response mainly depends on the properties of that vaccine [10,14]. It has become evident that an antigen delivered to the DCs in a particulate form can generate a Th1-type response [15]. Furthermore, the results suggest that the adjuvanted particulate vaccine is cross presented via the MHC I and II pathways, as seen by the increased expression of MHC I and MHC II and their co-stimulatory molecules.

The skin is an excellent site for vaccine administration as it consists of several Langerhans cells and dendritic cells, which are APCs capable of engulfing the microparticulate vaccine activating the sequence of events involved in the generation of an antigen-specific immune response [35,53]. Dissolving microneedles, as the name suggests, quickly dissolve to release the vaccine MP into the immune-rich layers of the skin [29,35]. Thus, to further corroborate the results obtained by in vitro testing, we also assessed the vaccine MP in vivo in mice administered using dissolving microneedles. The mice sera were collected and evaluated for antibody levels following immunization.

Serum antibodies are required to prevent the entry of pathogens by binding to them, thereby preventing their binding to the host cells in the body [19,54]. The induction of a humoral (antibody-mediated) response is one of the most critical requirements for vaccine development [55]. Particularly IgG and IgA play a vital role in antigen binding and neutralization, thus preventing viral infection of the host cells [54,56]. The iCCoV MP induced high antigen-specific serum IgG antibodies in mice for all weeks of serum analysis compared to the No treatment group. Serum IgA, however, was only significantly high during week four after the first booster dose. Typically, serum IgA dominates in the early weeks of vaccination, and the response obtained is a function of the antigen and the route of vaccine administration [56,57,58]. A mucosal route of vaccination may induce better secretory and serum IgA responses following vaccination [59,60]. It is evident from the data obtained that microneedle administration results in increased levels of serum IgG over IgA.

Further, we subtyped the IgG responses to understand better the role of IgG in inducing a Th1 or Th2 type response. Typically, increased IgG1 levels are associated with a Th2-biased helper T cell response, whereas IgG2a levels are associated with a Th1-biased cytotoxic T cell response [52,61]. We observed that the IgG1 levels of the adjuvanted MP vaccine peaked at week six and remained high during week 8. The results suggest that helper T cell responses may increase after administering two doses of the vaccine. The IgG2a level of the adjuvanted vaccine was significantly higher than the No treatment group during week two and week six. The IgG subtyping reveals that IgG1 responses are higher than the IgG2a responses, suggesting that helper T cell responses may dominate the cytotoxic T cell responses. These results can further be supported by assessing the cell-mediated immune responses produced following vaccination.

To summarize, we found that the adjuvanted microparticulate vaccine effectively induced an innate immune response in vitro compared to the antigen in suspension form. Our dual delivery system of a microparticulate vaccine-loaded in a dissolving microneedle array can increase antibody levels in immunized mice. Furthermore, microneedles are pain-free and can be self-administered, which is an attractive option for mass vaccinations during a pandemic [29,35]. Moreover, the adjuvants (Alhydrogel^®^ + AddaVax™), when combined with the iCCoV MP, greatly enhanced the immune response in vitro and in vivo. Assessing antigen-specific antibody levels in a preclinical animal model is a critical step in vaccine development. The data shown indicate that MN vaccination increases antibody responses in mice. Our future studies will focus on evaluating and characterizing cell-mediated responses induced by the adjuvanted vaccine MP. 

## 5. Conclusions

The microparticulate vaccine delivery system and the dissolving MN for vaccine administration were tested using the iCCoV as a model antigen. The MP form of the vaccine was found to be more immunogenic than the antigen suspension in vitro. The addition of adjuvant MP Alhydrogel^®^ and AddaVax™ significantly increased the immunogenicity of the MP vaccine in vitro. The antigen and adjuvant MP were found to be non-cytotoxic to cells in vitro. The adjuvanted vaccine MP induced a significant expression of antigen-presenting molecules and their co-stimulatory molecules on the surface of DCs. Cross-presentation of the antigen by the DCs was another key observation. In vivo, the vaccine effectively induced elevated antibody levels in vaccinated mice and will be further assessed for its ability to activate cell-mediated immunity.

## Figures and Tables

**Table 1:** Description of treatment groups involved in the in vivo study.

	Treatment Groups for In Vivo Study (*n* = 4)		
No.	Group	Description	Dose of Antigen/Adjuvant
1	Naive	No treatment	N/A
2	iCCoV suspension	Inactivated CCoV antigen suspension in MN	20 μg antigen suspension
3	iCCoV MP	Inactivated CCoV PLGA MP in MN	MP equivalent to 20 μg antigen
4	(iCCoV + Alhydrogel^®^ + AddaVax™) MP	(Inactivated CCoV PLGA MP + Alhydrogel^®^ PLGA MP + AddaVax™ PLGA MP) in MN	MP equivalent to (20 μg iCCoV + 30 μg Alhydrogel^®^ + 5 μg AddaVax™)

**Table 2:** Characterization results of vaccine and adjuvant MP.

S.No	Parameter	Mean ± Standard Deviation (SD)		
		iCCoV MP	Alhydrogel^®^ MP	AddaVax™ MP
1.	Product yield (%)	91 ± 5	93 ± 5	90.5 ± 5
2.	Particle size (nm)	809.2 ± 209.8	1573 ± 278.6	1274 ± 259.1
3.	Zeta potential (mV)	−15.4 ± 2.31	12 ± 0.252	−12.5 ±2.65
4.	PDI	0.70 ± 0.036	0.954 ± 0.04	0.896 ± 0.069

## Data Availability

Data will be made available upon reasonable request.
